# State-of-the-art technologies for the digital transformation of healthcare services – a systematic scoping review

**DOI:** 10.1186/s12913-026-14714-9

**Published:** 2026-06-27

**Authors:** Thomas C. Richards, Ji Han, Saeema Ahmed-Kristensen

**Affiliations:** 1 Department Innovation, Technology, Entrepreneurship, University of Exeter Business School, Faculty of Environment, Science, Economy, Streatham Campus, University of Exeter, Exeter, EX4 4PU UK; 2 NIHR Exeter Biomedical Research Centre, Medical School, St Luke’s Campus, University of Exeter, Exeter, EX1 2LU UK

**Keywords:** Digital transformation, Digital technologies, Healthcare services, Health systems design, Systematic review, Industry 4.0, Artificial intelligence, Big data, Internet of things, Blockchain

## Abstract

**Background:**

The implementation of digital technologies within health services promises increased performance, quality, and efficiency. However, evidence is limited regarding the application and outcomes of digital technologies implemented at the level of health services (meso-level) or health systems (macro-level), rather than those evaluated solely as patient-level clinical interventions (micro-level). We systematically review state-of-the-art digital technologies and their applications in healthcare systems and conduct a framework synthesis to review outcomes related to maturity and implementation.

**Methods:**

Eleven databases were searched (Embase, HMIC, Medline, PsycInfo, SPP, AgeLine, AMED, CDAS, CINAHL, SCOPUS, WoS) on 4th November 2024 for systematic and non-systematic reviews published within the previous five years that provided an overview of digital technologies applied at a systems/service-level (macro/meso-level) in healthcare or evidence for their outcomes. Studies into individual interventions (micro-level) were excluded. Risk of bias/quality assessment tools used in the reviews were recorded. We review types and applications of technology, then use a framework synthesis methodology to assess outcomes relating to digital transformation awareness/maturity, implementation, UK context, and challenges.

**Results:**

Our searches identified 1423 records, with 1011 remaining after deduplication. Of these, 131 were assessed for eligibility, with 28 reviews reporting on 1606 individual studies (with an additional 2437 included in a bibliometric analysis) included in the final analysis. We identified five main groups of technology in healthcare: Integrated Technology/Industry 4.0, Artificial Intelligence (AI), Big Data, Internet of Things (IoT), and Blockchain. Our framework synthesis revealed recent conceptual recognition of digital transformation, and variability across regions with regards to the sophistication and maturity of uptake of technologies. We report on four reviews which provided quantitative evidence on the impact of digital technologies in health services. Barriers included major concerns related to data privacy and trust, equity, ethics, and implementation logistics. UK frameworks exist for implementation of technologies, but do not focus exclusively on the systems-level.

**Conclusions:**

Digital technologies are increasingly integrated into health service delivery, yet the maturity and evaluation of these tools vary across regions. More robust implementation research is needed for advanced systems. Our synthesis provides a framework for healthcare leaders to assess digital readiness and prioritise technologies aligned with service transformation goals. Limitations of evidence include the heterogeneity of technologies, settings, and outcomes across included reviews.

**Trial Registration:**

The review was not part of a trial and was not registered.

**Supplementary Information:**

The online version contains supplementary material available at https://doi.org/10.1186/s12913-026-14714-9.

## Background

Healthcare systems worldwide struggle to meet challenges of healthcare delivery and equity against the backdrop of restricted resources, an aging population, and complex healthcare needs [1]. As the number of people diagnosed with multiple long-term conditions increases due to factors such as longer life expectancy [2], healthcare systems structured to address single diseases duplicate interactions [3], affecting care outcomes and adding strain to system resources. There is a clear need to restructure services to increase efficiency and reduce costs, while preserving patient experience and health outcomes. Healthcare commissioners are turning to digital technologies to meet these needs, though their impact is variable [4].

Interest in healthcare systems’ adoption of digital technologies is proliferating. This is well-illustrated by a review on the topic which identified an increase from 26 to 188 relevant papers over the years 2020 and 2021 alone [5]. This expansion reflects a shift from basic digitisation efforts to broader organisational change initiatives. For the context discussed herein, it is important to differentiate between three often confused concepts: digitisation, digitalisation, and digital transformation. The terms digitisation and digitalisation refer to the conversion of records from analogue to digital, and the integration of digital technologies into healthcare operations, respectively [6]. Digital transformation, however, refers to a fundamental, system-level reconfiguration of how healthcare services are organised and delivered. It is enabled by digital technologies but extends beyond tools themselves. It encompasses changes in workflows, roles, organisational culture, service models, and value creation within health systems [7–9]. In this paper, we adopt definitions used in the broader health systems literature, where digital transformation is conceptualised as a holistic, system-level process of organisational and service redesign supported by digital technologies, aimed at enabling new or improved models of care [7, 8]. We use the term system-level to refer to technologies that influence the organisation, management, or delivery of healthcare services or health systems (meso- or macro-level impacts), rather than technologies evaluated solely as interventions at the level of individual patients (micro-level impacts).

Policymakers have increasingly endorsed digital transformation as a route to modernising health systems. For example, in the UK, the NHS Long Term Plan identifies digital technology as central to enabling more integrated, responsive, and sustainable care [10]. Meanwhile, the COVID-19 pandemic significantly accelerated the uptake of remote consultation tools, exposed limitations in digital infrastructure, and increased momentum for long-term digital reform.

Despite growing policy support, the evidence base on system-level applications of digital technologies, which underpins the digital transformation of healthcare, remains fragmented. Numerous reviews have focused on specific technologies such as telemedicine [11, 12], mobile apps [13, 14], or artificial intelligence [15–19]. However, much of this literature is out of date considering recent technology uptake [11], focused on patient-level health outcomes [14], specific conditions or technologies [13], or user perspectives [12]. There is also a lack of evidence that maps the technologies applied in relation to their effects at the service or systems level, such as effects on quality of care outcomes within individual services, on management within healthcare organisations, through to effects of mass technology adoption within healthcare systems. Research is required to fill these gaps and determine what outcomes technologies deliver, and which are most mature or promising for large-scale implementation.

This paper addresses these gaps with the research questions: (1) what state-of-the-art digital technologies are driving system-level transformation in healthcare, and in what contexts and applications; and (2) how does the literature conceptualise the digital transformation and present evidence for implementation, maturity, outcomes, and barriers to large-scale adoption for identified technologies? To address these questions, we conducted a systematic scoping review, synthesising evidence from existing literature reviews and systematic reviews of digital technologies impacting health services or systems. We combined this with a best-fit framework synthesis, using an a priori framework to organise the evidence around conceptual, contextual, and implementation domains.

We take a data-driven approach to describe technologies for research question 1), using the definitions provided above to inform both our inclusion criteria and analytic framework. In a two-stage screening process, we first shortlist reviews of technologies with the potential to drive service- or system-level change, consistent with the concept of digital transformation. In the final stage we screen out those reporting directly on technologies for digitisation and digitalisation, aiming to focus on reviews reporting state-of-the-art technologies. We analyse how these technologies might interact with workforce practices, workflows, organisational readiness and contextual constraints using elements from System Engineering Imitative for Patient Safety (SEIPS) 2.0 socio-technical systems framework [20] as a theoretical lens. To address question 2), our best-fit framework synthesis was structured to capture concepts central to digital transformation, including readiness, implementation maturity, service-level outcomes, and barriers to scale-up.

The approach used in this review enabled us to identify major technology groups contributing to digital transformation, summarise their applications, and assess reported outcomes and barriers to implementation. The findings are intended to support service designers, policymakers, and digital health strategists in planning effective and evidence-informed transformation efforts. We synthesize international evidence so that learning can be applied within the context of NHS policy towards digital transformation in the UK. The UK context is addressed as one domain of our analytical framework.

## Methods

### Study design

We conducted a systematic scoping review in combination with a best-fit framework synthesis restricted to review-level evidence. Scoping reviews are well suited to mapping broad themes and can synthesise evidence from diverse sources including systematic and non-systematic reviews [21]. This approach aligns well with our aims since non-systematic reviews (such as narrative reviews or literature reviews) can describe the range of technologies available relevant to research question 1, whereas systematic reviews provide a benchmark for evaluating their implementation for research question 2. The best-fit framework synthesis method (see section “Framework synthesis” section below) allows evidence to be synthesised against an a priori conceptual framework [22], which supports the analysis required to address research question 2 as stated in the section “Background”. This review was conducted in accordance with the PRISMA extension for scoping reviews (PRISMA-ScR) [23]. A protocol was developed prior to the review but was not registered.

### Eligibility criteria

We included secondary research studies (reviews, narrative reviews, systematic reviews, overviews, meta-analyses, and meta-syntheses) published within the five years preceding the search date (4th November 2019–4th November 2024) that focused on the application of digital technologies at a systems or service level within healthcare contexts. This timeframe was selected to ensure inclusion of the most recent technological developments. The search was restricted to articles written in English. Non-systematic reviews (such as narrative or descriptive reviews that do not employ a structured or reproducible search and selection process) were included if they presented lists of technologies which have impacts on a healthcare system or service at any level, from individual services through to management of healthcare systems. Primary studies, reviews without a healthcare setting, and reviews focused solely on individual patient-level interventions without reference to systemic or organisational impacts were excluded. No geographical restrictions were applied to where research originated from so that learnings could be extracted from a diversity of healthcare ecosystems.

### Information sources

The following electronic databases were searched on 4th November 2024:

1. Ovid: Embase, Health Management Information Consortium (HMIC), Medline, PsycInfo, Social Policy and Practice (SPP).
2. EBSCO: AgeLine, AMED - The Allied and Complementary Medicine Database, Child Development & Adolescent Studies (CDAS), CINAHL (Cumulative Index to Nursing and Allied Health Literature) Ultimate.
3. SCOPUS.
4. Web Of Science: Web of Science Core Collection.

### Search strategy

The search strategy was developed using the PICo framework (Population, phenomenon of Interest, Context) [21], tailored for each database, and included controlled vocabulary terms and free text keywords relating to healthcare system (re)design (P), digital transformation/technology (I), and review study types (Co) (Table 1). In the PICo framework, Context (Co) commonly refers to setting (e.g., primary care, hospitals, or specific countries). However, the JBI Manual for Evidence Synthesis [21] states “In a qualitative review, context will vary depending on the objective of the review.” In our review, setting was unrestricted and encompassed within the “Population” term (any healthcare system or service). We therefore utilised Context to describe study design, as this best represents the context in which relevant evidence is generated. Search terms were adapted from existing reviews on topics related to “(Re)design [24] of Health Systems [25] /Services [24]”, “Digital Transformation [26]”, as well as other terms added manually by the authors. For “Study Design”, we adapted existing search filters for reviews [27]. The full search strategy and results are provided in Additional File 1 - Search Strategy and Results.docx.

**Table 1:** PICo items

*P*	Population	Healthcare Systems/Service Design/Redesign
I	Phenomenon of interest	Digital Transformation/Digital Technology
Co	Context	Study Design

P, Population; I, Phenomena of Interest, Co, Context

For Phenomena of Interest (I), the key search terms are summarised in Table 2 (note that this table is a representative example using British English – for the full search strategy, which includes wildcards for US spelling variants, please see Additional File 1 - Search Strategy and Results.docx).

**Table 2:** Key search terms for phenomena of interest

Digital transformation
Digital
Digitalisation
Digitisation
Digital Shadow
Digital Twin
Symbiotic Simulation
Realtime Simulation
Big Data
Realtime Data
Cloud Computing Data
Sensor Data
(Industrial) Internet of Things (IoT)

IoT, Internet of Things

During preliminary scoping, we also developed searches, including relevant synonyms and methods, for artificial intelligence (AI) / machine learning (ML), and for computer modelling and simulation for inclusion within the Phenomena of interest term. These returned large numbers of hits (265134 for AI/ML, and 687185 for computer modelling on Ovid Medline, one of 11 databases explored) and will therefore be explored in separate studies. Further information regarding this preliminary scoping can be found in Additional File 1 - Search Strategy and Results.docx.

### Selection process

Search results were deduplicated using the Deduplicator tool [28], and screening and full-text appraisal were performed using the Screenatron and Disputatron tools [29]. A two-stage screening process was employed. Stage 1 consisted of identification of reviews on digitisation, digitalisation, and digital technologies. An overview of counts of technology types was produced. Stage 2 identified a focused subset for full-text review to select reviews addressing state-of-the-art technologies applied at systems level. Each stage was conducted independently by two reviewers who resolved disagreements through discussion. A PRISMA flow diagram was generated to show the search results and the process of screening and selecting studies for inclusion in the review. Studies excluded at full text stage were added to a table which includes the main reasons for their exclusion (Additional File 2 - Reasons for Exclusion.xlsx). The final number of studies included for screening from each database is presented in Table 3.

### Data extraction

First, characteristics of studies and counts of topics of focus of reviews was conducted. Data were extracted by one reviewer, which was then checked and developed if required by a second. Data extracted from the reviews included descriptive factors such as publication type, year of publication, country of origin, number of studies included in review (if systematic review), and contextual factors such as the reasons for conducting the review, the stated focus, the health focus, the application of the technology, and the geographical focus. The results are presented in (Table 4) . We use a data-driven approach is which is informed by the terminology used by the original authors, as we hypothesise that this might reflect subtle differences in intended meaning relating to, for example, for the type of technology or setting in which it is applied. Note throughout the manuscript and associated figures and tables, this results in some inconsistencies between British/American spellings due to direct or indirect quotation from source materials. We then conducted a data synthesis based on the technology applications, followed by a framework synthesis based on specific outcomes.

### Data synthesis

The data synthesis consisted of three analytical steps followed by a narrative review, which was used to address research question 1.

First, we used an inductive approach to group individual technologies extracted from reviews based on how they were described and applied, rather than imposing a predefined taxonomy. This allowed the synthesis to accommodate the heterogeneity of contexts and use-cases across studies, identify empirically grounded higher-level technology categories, and avoid vague or inconsistent technology characterisations often reported in titles and abstracts. Reviews and technologies could be included in multiple groups depending on their applications and scope, and were charted alongside their applications and healthcare focus. After deriving the inductive groups, we interpreted them using a bespoke functional technology framework, which defines digital technologies by their role within digital transformation (e.g., data capture, security and validation, data aggregation, analytics, and system-level implementation).

Second, the maturity of technologies was characterised within each group as experimental (early-stage development, or pilot testing), emerging (feasibility stages, early adoption, or limited deployment), or embedded (routine use or established integration into care) based on how they were portrayed in reviews. This served as a descriptive indicator of their real-world implementation readiness and appropriateness.

Third, to analyse their implications for digital transformation as a socio-technical process we interpreted each technology group using elements from the System Engineering Imitative for Patient Safety (SEIPS) 2.0 socio-technical systems framework [20]. We selected the SEIPS 2.0 framework as a well-established socio-technical model developed for healthcare contexts. It provides a structured approach to analysing interactions among people, tasks, technologies, organisational structures, and environments, as well as associated processes and outcomes. As a domain-specific instantiation of socio-technical systems theory [30, 31], SEIPS is particularly well suited to examining how changes, including the introduction of digital technologies, influence healthcare service delivery and system functioning. SEIPS was used analytically to highlight how the technologies reported in the reviews might interact with workforce practices, workflows, organisational readiness and contextual constraints.

These results were integrated into a structured table (Table 5) and reported alongside key use-cases using a narrative approach for each group.

### Framework synthesis

We used best-fit framework synthesis [22], a sub-type of framework synthesis methodology [32], to synthesise specific outcomes to address research question 2. Best-fit framework synthesis provides a transparent approach for integrating heterogeneous review findings by using a predefined conceptual structure while still allowing the framework to be adapted as new concepts emerge from the data. This approach aligns with our objective to examine digital transformation as a multi-dimensional socio-technical process spanning conceptual, organisational, and implementation domains. An initial a priori framework was developed based on the review objectives, consisting of five outcome domains: (1) conceptualisation of digital transformation in health services, (2) technology readiness and implementation maturity, (3) reported outcomes of digital transformation, (4) digital transformation in the United Kingdom, and (5) barriers and challenges of digital transformation. The framework was refined minimally during synthesis. Reviews were classified according to relevant domains, and findings were coded and synthesised across framework categories using NVivo 14 to enhance transparency and auditability of the analytical process.

### Cooccurrence map

A co-occurrence map of key words from included review titles and abstracts was generated using VOSviewer (version 1.6.20) to visually represent the thematic focus of the literature.

### Risk of bias assessment

The risk of bias/quality assessment tools (if any) applied by each review was included as a column in the study characteristics table (Table 4)

## Results

### Review selection

Our searches identified 1423 records. There were 1011 unique records identified after deduplication (Table 3).

**Table 3:** Number of results retrieved from individual databases

	Ovid	EBSCO	SCOPUS	WoS	Total	Total (unique)
Number of results	629	115	493	186	1423	1011

**Fig. 1:**
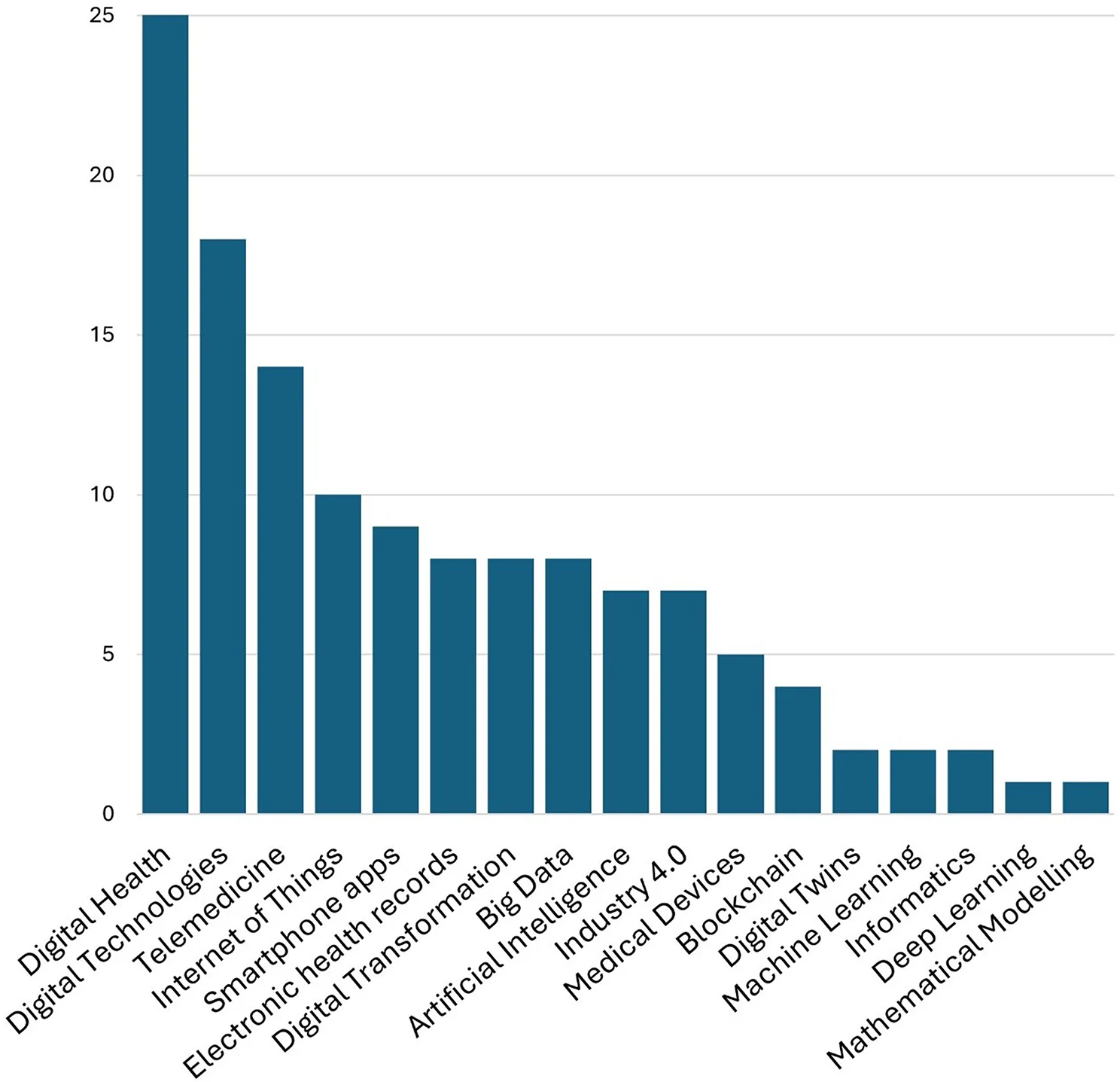
Counts of technology areas. Counts of technologies covered in the 131 reviews identified in stage 1 of screening

After stage 1 of the screening process, we grouped technologies to provide an overview of counts of technology areas, which included 131 papers (Fig. 1).

**Fig. 2:**
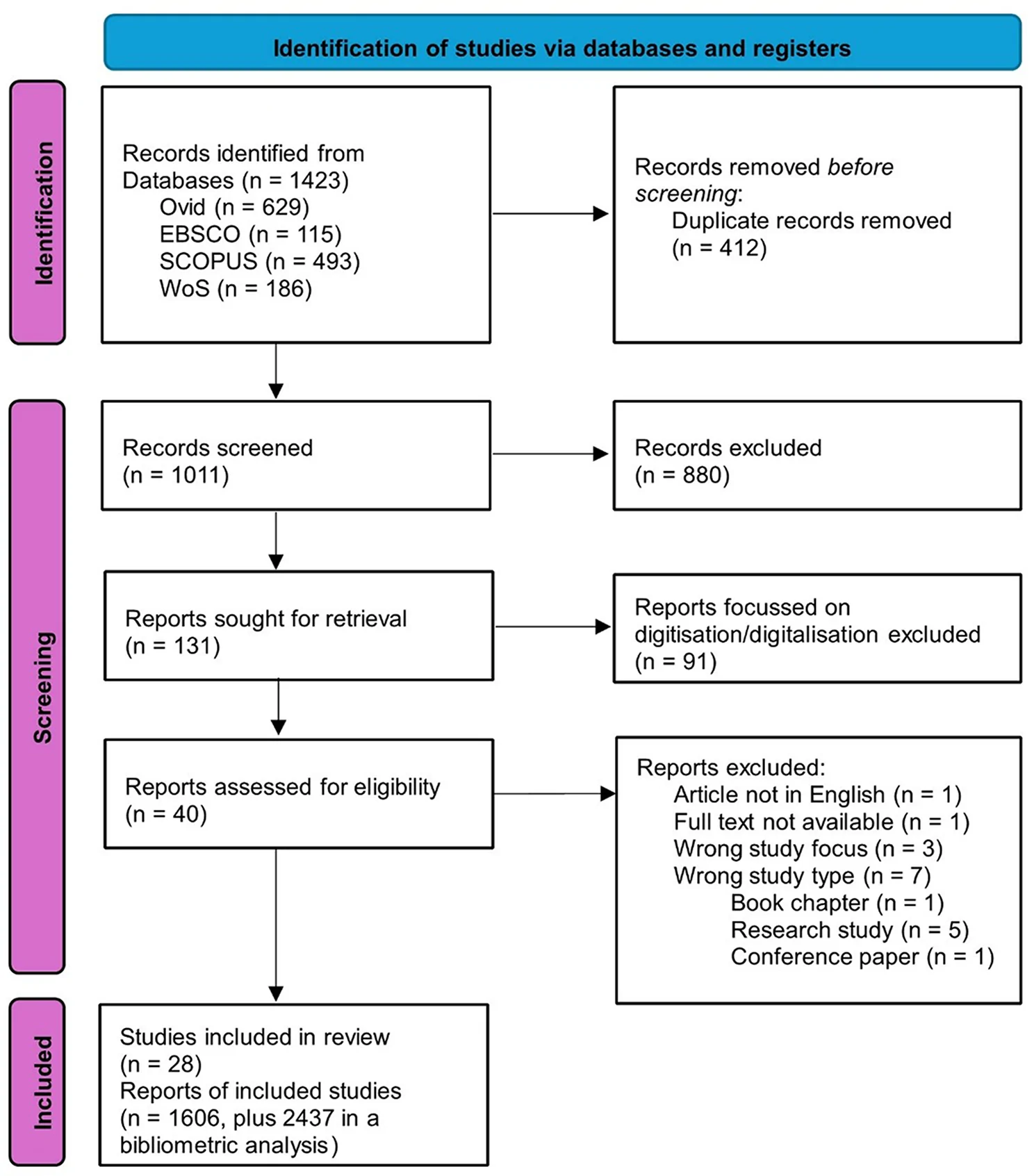
PRISMA flow diagram

For stage 2 of screening, 40 reviews were identified as eligible for full-text review. Of these, 28 reviews [33–60] were included after full-text review, covering a reported total of 1606 individual studies (with an additional 2437 included in a bibliometric analysis). Some reasons for exclusion were wrong study type, wrong study focus, study not in English, and manuscript not available. Furthermore, a number of studies which were excluded because they did not have sufficient focus on the service/systems-level impacts of technologies over patient-level interventions (e.g [61–63]). Figure 2 shows a PRISMA flow diagram illustrating the screening process. See Supplementary Material 3 - PRISMA-ScR-Fillable-Checklist_10Sept2019.docx for the PRISMA-ScR Checklist.

### Review characteristics

The characteristics of the reviews, including the risk of bias/quality assessment tools used, are summarised in Table 4.

#### Review types

We identified 17 Systematic Reviews and 11 Non-Systematic reviews. The Systematic Review types included Systematic Review (*n* = 7), Systematic Literature Review (*n* = 5), Scoping Review (*n* = 2), Systematic Review and Meta-Analysis (*n* = 1), Meta-Analysis (*n* = 1), and Cochrane Review (*n* = 1). The Non-Systematic Review types included Review (*n* = 9), Narrative review (*n* = 1), and Perspective (*n* = 1). Within the dataset, there were four papers which included quantitative data regarding the effects of digital technologies on health service outcomes, which we report on for the framework synthesis in relation to the domain: “Reported outcomes of digital transformation” [33, 38, 48, 59].

#### Setting

The reported healthcare context of the included reviews were Healthcare (*n* = 12), Healthcare Services (*n* = 5), Primary Healthcare (*n* = 3), NHS (*n* = 2), Healthcare Sector (*n* = 1), Secondary Care/Outpatient Services (*n* = 1), Healthcare Systems (*n* = 1), Mental Health Services (*n* = 1), Healthcare Organisations (*n* = 1), and Rural Healthcare Systems (*n* = 1).

#### Stated focus of reviews

The central foci of reviews stated in titles or abstracts were Digital Health Platforms (*n* = 7) [33, 46, 48, 54, 55, 57, 58], Digital Technologies (*n* = 7) [34–36, 38, 41, 50, 52], IoT (*n* = 5) [40, 45, 47, 53, 56], Big Data/Data Analytics (*n* = 5) [37, 43, 47, 49, 51], Industry 4.0 (*n* = 3) [35, 51, 60], AI/Deep Learning (DL) (*n* = 3) [44, 48, 56], Clinical Decision Support Tools (*n* = 2) [42, 59], and Blockchain (*n* = 1) [39]. Though terms for AI and computational modelling were excluded from specific search terms, some were captured under terms related to digital technologies. Digital Health Platforms and Digital Technologies are broad terms. Technologies covered within reviews focussing on Digital Health Platforms incorporated mobile phone-based technologies, or clinical digital platforms, including digital health apps [46, 57], mobile app-based interventions [48], clinical decision-making tools [33], as well as e-health innovations such as patient account, electronic prescriptions and electronic referrals [55, 64], and platforms which incorporate biotelemetry devices [58] or technologies used in home-based rehabilitation [54]. “Digital technologies” was used to refer by some authors to more mature m-health, e-health, telemedicine, and digital app platforms [34, 38, 41, 50]. However, it was also used to refer to emerging technologies such as AI, ML, and DL [34, 35, 50, 52], blockchain [34, 35], cloud computing [35], big-data analytics [35, 50, 52], IoT [34, 35, 52], virtual reality (VR)/augmented reality (AR) [52], health management information systems [36], and also genomics, analytics, and smart devices and wearables [50].

**Table 4:** Characteristics of the included reviews

Review Type	Author, Year	Title	Study Type	*N*. studies	Review aim	Stated Focus	Health Service Focus	Application of Technology	Geographical Focus	Risk of Bias / Quality Assessment methods
Systematic	Agarwal, et al., 2021	Decision-support tools via mobile devices to improve quality of care in primary healthcare settings	Cochrane Review	15	Assess effects of Digital Clinical Support Systems in Primary Care Settings	Digital Health Platforms	Primary Healthcare	Clinical Decision-Making	Not Restricted	Criteria outlined in the Cochrane Handbook for Systematic Reviews of Interventions (Higgins 2017) Guidance from the EPOC group for assessing randomized trials
	Akhtar, et al., 2022	Efficacy and pitfalls of digital technologies in healthcare services: A systematic review of two decades	Systematic Review	55	Review digital technologies in healthcare services and assess Efficacy and Pitfalls	Digital Technologies	Healthcare Sector	Not Restricted	Not Restricted	None
	Arntz, et al., 2023	Technologies in Home-Based Digital Rehabilitation: Scoping Review	Scoping Review	RQ1 (bibliometric analysis): 2437 RQ2: 95 RQ3: 40	Identify digital technologies for home-based digital rehabilitation (DR), predict new or emerging DR trends, and report on the influences of the COVID-19 pandemic on DR.	Digital Health Platforms	Secondary Care Services Outpatient Services	Rehabilitation Disease Management Disease Monitoring	Not Restricted - Grey literature for Governments in Greece, Spain, Finland, Germany, and England	None
	Bolhasani, et al., 2021	Deep learning applications for IoT in health care: A systematic review	Systematic review	44	Conceptual and taxonomy of deep learning for IoT applications in healthcare systems reviewed, then advantages, disadvantages, and limitations discussed.	Artificial Intelligence (AI)/Deep Learning (DL), IoT	Healthcare Systems	Diagnosis Disease Monitoring Disease Prediction Behaviour	Not Restricted	None
	Buzancic, et al., 2024	Do clinical decision support tools improve quality of care outcomes in the primary prevention of cardiovascular disease: A systematic review and meta-analysis	Systematic Review and Meta-Analysis	16	Assess the effectiveness of Clinical Decision Support Tools (CDSTs) in enhancing the quality of care outcomes in primary cardiovascular disease (CVD) prevention.	Clinical Decision Support Tools	Primary Healthcare	Quality of Care Prevention	Not Restricted	Cochrane RoB-2 tool
	Cavallone, et al., 2020	Debunking the myth of industry 4.0 in health care: insights from a systematic literature review	Systematic Literature Review	40	To shed light on the applications, aftermaths and drawbacks of industry 4.0 in health care, and summarise the state of the art.	Industry 4.0	Healthcare	Quality of Service Service Effectiveness	Not Restricted	None
	Fanelli, et al., 2023	Big data analysis for decision-making processes: challenges and opportunities for the management of health-care organizations	Systematic Literature Review	48	To provide a picture of the current state of art in the use of big data for decision-making processes for the management of health-care organizations.	Big Data/Data Analytics	Healthcare Organisations	Organisation Management	Not Restricted	None
	Farre, et al., 2023	Exploring the use of digital technology to deliver healthcare services with explicit consideration of health inequalities in UK settings: A scoping review	Scoping Review	11	To map and explore existing evidence on the use of digital technology to deliver healthcare services with explicit consideration of health inequalities in UK settings.	Digital Technologies	NHS	Health Service Delivery Targeted Service User Communication Health Monitoring Decision Support Systems Telemedicine	United Kingdom	None
	Fatoum, et al., 2021	Blockchain integration with digital technology and the future of health care ecosystems: Systematic review	Systematic Review	8	To explore blockchain technology, its framework, current applications, and integration with other innovations, as well as opportunities in diverse areas of health care and clinical research, in addition to clarifying its future impact on the health care ecosystem.	Blockchain	Healthcare	Disease Monitoring Disease Management Diagnosis Evaluation	Not Restricted	None
	Ge, et al., 2023	A systematic and comprehensive review and investigation of intelligent IoT-based healthcare systems in rural societies and governments	Systematic Review	17	To conduct an extensive review of intelligent IoT-based healthcare systems in rural communities and their governance.	IoT	Rural Healthcare Systems	Disease Monitoring	Not Restricted	Quality Evaluation Checklist
	Haque, et al., 2022	Semantic Web in Healthcare: A Systematic Literature Review of Application, Research Gap, and Future Research Avenues	Systematic Literature Review	65	To assess and critique previous findings related to Semantic Web Technology.	Clinical Decision Support Tools	Healthcare	Information Management	Not Restricted	None
	Nasarian, et al., 2024	Designing interpretable ML system to enhance trust in healthcare: A systematic review to proposed responsible clinician-AI-collaboration framework	Systematic Review	74	To review the processes and challenges associated with interpretable machine learning (IML) and explainable artificial intelligence (XAI) within the healthcare and medical domains.	AI/DL	Healthcare	Clinical Decision-Making	Not Restricted	Meta Quality Appraisal Tool (MetaQAT)
	Saheb, et al., 2019	Paradigm of IoT big data analytics in the healthcare industry: A review of scientific literature and mapping of research trends	Systematic Literature Review	130	To identify how the IoT Big Data Analytics paradigm has impacted the design, development, and application of IoT based innovations in healthcare services.	IoT Big Data/Data Analytics	Healthcare Services	Analytics Management	Not Restricted	Study Quality Assessment: included if had a real case study related to analytics in health domain
	Saif-Ur-Rahman, et al., 2023	Artificial intelligence and digital health in improving primary health care service delivery in LMICs: A systematic review	Systematic Review	48	To explore the evidence on the use of AI and digital health in improving primary health care service delivery. Methods:	AI/DL Digital Health Platforms	Primary Healthcare Services	Improvement of Service Delivery	Low and Middle Income Countries (LMICs)	Quality assessment tools provided by the Joanna Briggs Institution for cross-sectional studies, analytical studies, quasi-experimental studies, and experimental studies Mixed-methods appraisal tool (MMAT) for Mixed-methods studies
	Salazar-Reyna, et al., 2022	A systematic literature review of data science, data analytics and machine learning applied to healthcare engineering systems	Systematic Literature Review	576	To assess and synthesize the published literature related to the application of data analytics, big data, data mining and machine learning to healthcare engineering systems.	Big Data/Data Analytics	Healthcare	Analytics	Not Restricted	None
	Sisodia, et al., 2021	A meta-analysis of industry 4.0 design principles applied in the health sector	Meta-Analysis	43	A meta-analytic approach to investigate the findings of original articles into big data and industry 4.0 together for healthcare.	Industry 4.0 Big Data/Data Analytics	Healthcare	Health Management Health Monitoring Digitalisation of Services	Not Restricted	Quality assessment checklist: proposed techniques, novelty, and the methodology used by them in the context of dataset and methods used for evaluation
	Zahid, et al., 2021	A systematic review of emerging information technologies for sustainable data-centric health-care	Systematic Review	69	To review emerging information technologies for data modelling and analytics that have potential to achieve Data-Centric Health-Care (DCHC) for the envisioned objective of sustainable healthcare.	Digital Technologies	Healthcare	Analytics Modelling	Not Restricted	None
Non-Systematic	Al-Shammri, et al., 2024	Developing Healthcare using Internet of Things (IoT): A Survey of Applications, Challenges and Future Directions	Review	NS	Survey of Applications, Challenges and Future Directions of IoT in Healthcare	IoT	Healthcare Services	Disease Management Disease Monitoring Compliance Monitoring	Not Specified	None
	Bialczyk, et al., 2024	Exploring Digital Health Horizons: A Narrative Review of E-Health Innovations in Poland, Spain, Romania and Estonia	Narrative review	NS	Overview of e-health innovations in Poland, Spain, Romania and Estonia, focussing on the digital development of healthcare services.	Digital Health Platforms	Healthcare Services	Digitalisation of Healthcare Services eHealth	Poland, Spain, Romania and Estonia	None
	Bond, et al., 2023	Digital transformation of mental health services	Perspective	NS	Discuss challenges of digital health for mental health services	Digital Health Platforms	Mental Health Services	Diagnosis Disease Monitoring Disease Prediction Behaviour	Not Specified	None
	Busnatu, et al., 2022	A Review of Digital Health and Biotelemetry: Modern Approaches towards Personalized Medicine and Remote Health Assessment	Review	NS	To review the concepts correlated to digital health, classify and describe biotelemetry devices, and present the potential of digitalization for remote health assessment, the transition to personalized medicine, and the streamlining of clinical trials.	Digital Health Platforms	Healthcare Services	Remote Assessment	Not Restricted	None
	Chandra, et al., 2022	Digital technologies, healthcare and Covid-19: insights from developing and emerging nations	Review	NS	To emphasises the usefulness, most recent development, and implementation of Digital technologies, Industry 4.0 techniques, and tools in fighting the COVID-19 pandemic worldwide. Keywords	Digital Technologies Industry 4.0	Healthcare	Disease Prevention Telemedicine	Emerging nations	None
	Dahiya, et al., 2022	Designing Delivery of Healthcare Services with Health Management Information System, Artificial Intelligence, Big Data, and Innovative Digital Technologies	Review	NS	To understand how Health Management Information Systems (HMIS) can be used to design health- care delivery using advanced, ingenious, and revolutionary technologies such as AI, Innovative Digital Technologies (IoT), and Big Data.	Digital Technologies	Healthcare Services	Health Records	India (Delhi-NCR), America, Asia, and Africa	None
	Hameed, et al., 2024	Digital transformation for sustainable health and well-being: a review and future research directions	Review	15	To provide a research literature study covering an important sustainable development goal (SDG), “Good health and well-being,” and its digital transformation, along with summarising research findings in a detailed and taxonomical way.	Digital Technologies	Healthcare	Digitalisation	Not Restricted	Quality Evaluation Checklist
	Karatas, et al., 2022	Big Data for Healthcare Industry 4.0: Applications, challenges and future perspectives	Review	82	To review publications which lie within the intersection of Industry 4.0, Big data, and healthcare operations and give future perspectives.	Big Data/Data Analytics	Healthcare	Management	Not Specified	None
	Peek, et al., 2023	Digital health and care: Emerging from pandemic times	Review	NS	To describe how disruption of health and care services caused by the COVID- 19 pandemic and the rapid changes in digital service delivery, artificial intelligence and data sharing, have progressed since 2020, reflect on lessons learnt and consider key challenges and opportunities ahead by reviewing significant developments reported in the literature.	Digital Health Platforms	NHS	Digitalisation	UK Focus	None
	Senbekov, et al., 2020	The recent progress and applications of digital technologies in healthcare: A review	Review	152	To discuss and analyse the recent progress on the application of big data, artificial intelligence, telemedicine, block-chain platforms, smart devices in healthcare, and medical education. Basic	Digital Technologies	Healthcare	Disease Monitoring Diagnostics Digitalisation of Healthcare	Not Restricted	None
	Zeadally, et al., 2020	Smart healthcare: Challenges and potential solutions using internet of things (IoT) and big data analytics	Review	NS	To identify some of the challenges that need to be addressed to accelerate the deployment and adoption of smart health technologies for ubiquitous healthcare access.	IoT	Healthcare	Service Improvement Analytics	Not Specified	None

RQ, Research Question; DR, Digital Rehabilitation; IoT, Internet of Things; AI, Artificial Intelligence; DL, Deep Learning; CDST, Clinical Decision Support Tool; CVD, Cardiovascular Disease; RoB, Risk of Bias; NHS, National Health Service; MetaQAT, Meta Quality Appraisal Tool; IML, Interpretable Machine Learning; XAI, Explainable Artificial Intelligence; LMIC, Low and Middle Income Country; MMAT, Mixed-Methods Appraisal Tool; DCHC, Data-Centric Health-Care; NS, Not Specified; HMIS, Health Management Information System; SDG, sustainable development goal

### Data synthesis: state-of-the-art technologies and their applications

**Fig. 3:**
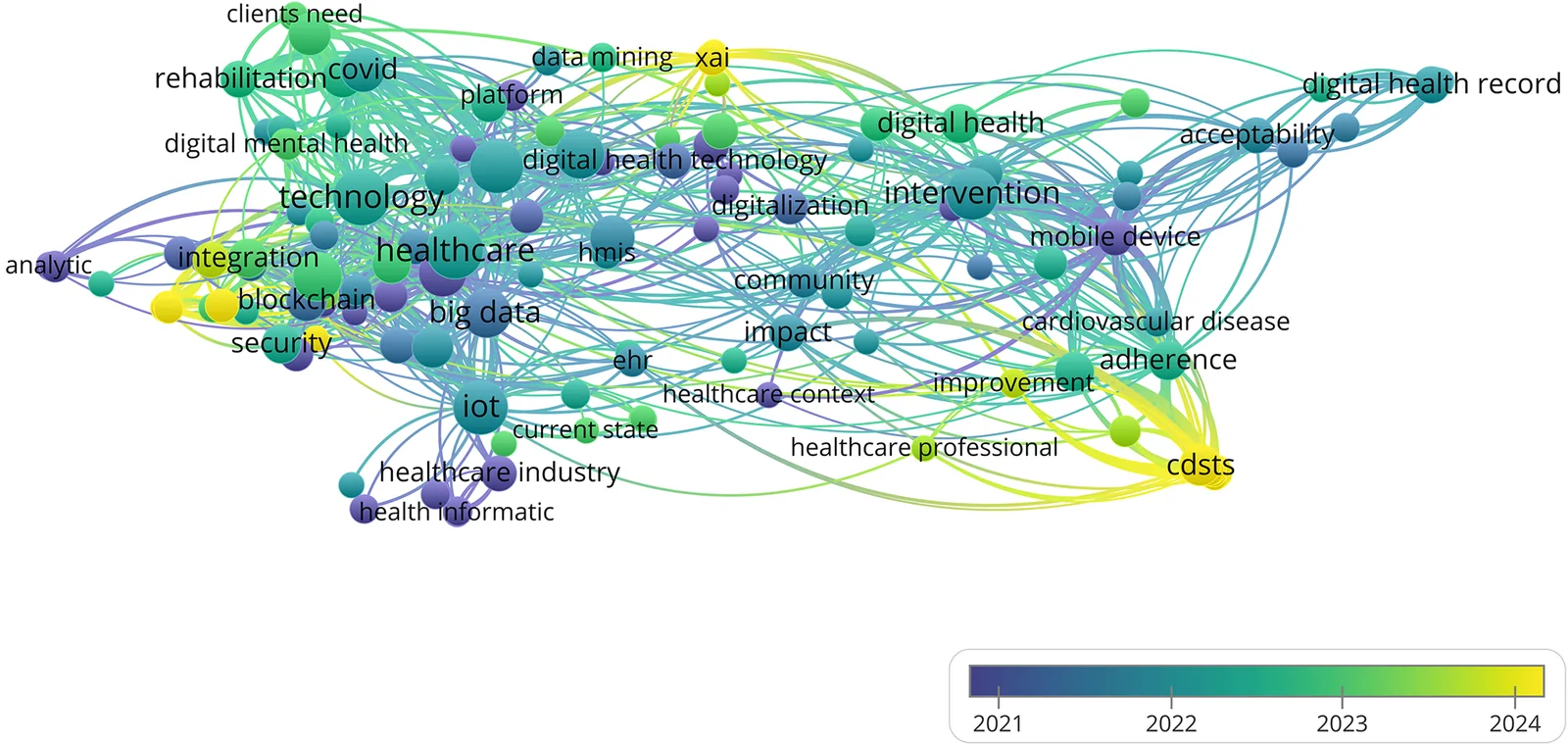
Co-occurrence network. Co-occurrence network of terms related to digital technologies within the included review titles and abstracts. XAI, Explainable Artificial Intelligence; EHR, Electronic Health Record; IoT, Internet of Things; CDSTs, Clinical Decision Support Tools

**Fig. 4:**
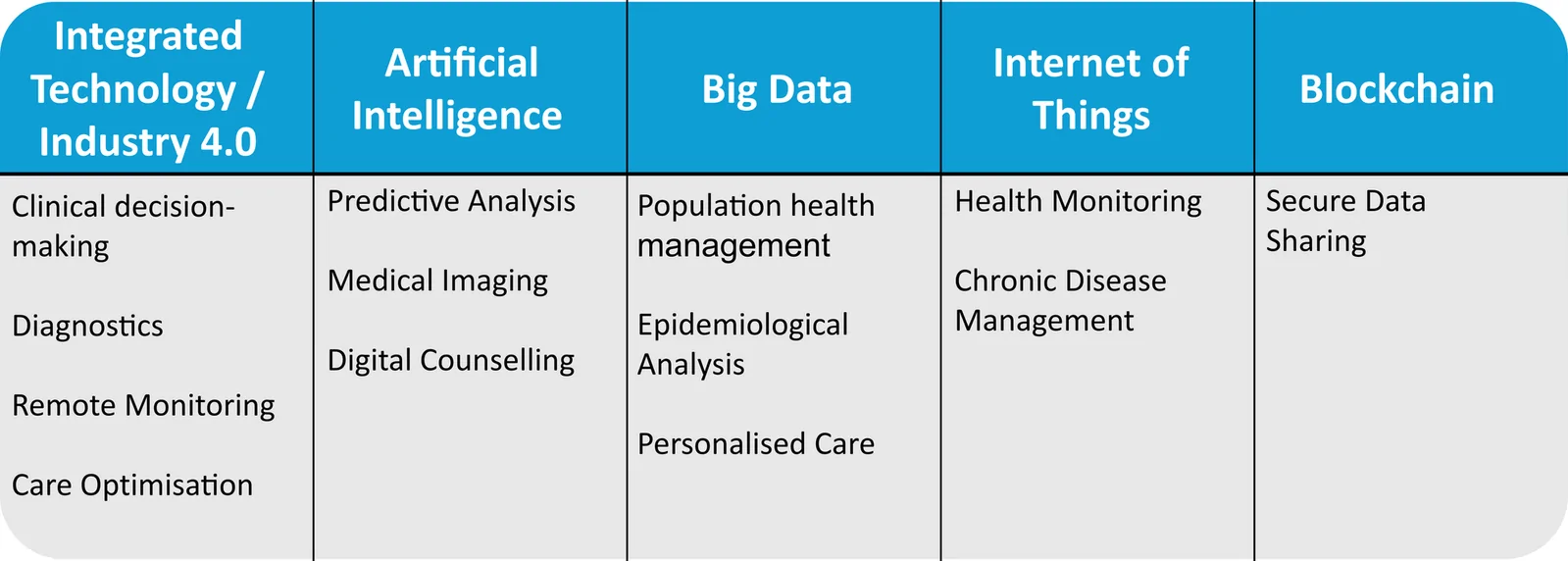
Summary of technology applications. The five technology groups identified and a summary of their main applications

Our inductive synthesis resulted in five groups: Integrated Technology/industry 4.0 (*n* = 12) [33–35, 38, 41, 50–52, 54, 55, 57, 60], AI (*n* = 5) [44, 46, 48, 56, 58], Big Data (*n* = 7) [37, 42, 43, 47, 49, 51, 59], IoT (*n* = 5) [40, 45, 47, 53, 56], and Blockchain (*n* = 2) [39, 41]. Boundaries between groups occasionally overlapped, reflecting how technologies were actually conceptualised and applied across the included reviews. To support interpretation, these groups were classified within a bespoke functional technology framework that defines digital technologies by their role within digital transformation. Within this framework, IoT corresponded to data capture; Blockchain to security and validation; Big Data tools to data aggregation; AI to data analytics; and Integrated/Industry 4.0 technologies to system-level implementation and coordination. Table 5 provides a structured summary of the groups, presenting the individual technologies discussed, the application and focus of the technologies, and the reported healthcare contexts alongside an appraisal of maturity and socio-technical analysis. Figure 3 shows co-occurrence of terms within the titles and abstracts of included reviews. Figure 4 summarises the key technologies and their applications.

#### Integrated technology/industry 4.0

There were 12 reviews which covered Integrated Technology/Industry 4.0 applications in healthcare [33–35, 38, 41, 50–52, 54, 55, 57, 60]. Industry 4.0 was discussed in the context of applications in healthcare [60], insights for digital technologies and healthcare from COVID-19 in developing nations [35], and a meta-analysis of Industry 4.0 design principles applied in the health sector [51]. Integrated technologies covered a broad range. More mature technologies included were platform/smartphone-based m-health, e-health, telemedicine, and digital apps [34, 38, 41, 50], such as digital health apps [57], clinical decision-making tools [33], e-health patient account, electronic prescriptions and electronic referrals [55]. However, some reviews covered the integration of emerging technologies within healthcare, including digital twins [52], AI, ML, and DL [34, 35, 50, 52], blockchain [34, 35], cloud computing [35], big-data analytics [35, 50, 52], IoT [34, 35, 52], VR/AR [52], smart devices [50, 54], and also genomics, analytics, and smart devices and wearables [50].

Digital twins are a state-of-the-art technology that have only relatively recently been incorporated within the healthcare ecosystem. They are able to integrate with other technologies such as AI or IoT to provide smart health applications. The general concept of the Digital twin was proposed in 2003 but has only recently been applied for healthcare tracking and monitoring. Zahid et al. (2021) describe Digital Twins in their review of emerging information technologies for sustainable data-centric healthcare [52]. Digital Twins use multi-physics, multi-science, and multi-scale models to provide rapid, efficient and accurate healthcare tracking and monitoring. A digital twin for healthcare consists of three parts: a physical object (patient, wearable device, intervention, or system containing all), a virtual object (digital person model, wearable device model, medical device model, digital system model or external factor model), and health data (real-time monitoring data from wearable device, detection data, medical records, or historical data). Zahid et al. (2021) [52] cite three characteristics of digital twins to be Real-Time Reflection, Interaction and Convergence, and Evolution and Iteration. The review identified contexts in which digital twins were applied to include monitoring and predicting health aspects of individuals from wearable devices, cardiac arrest detection using convolutional neural networks (CNNs), personalised healthcare, precision healthcare, a clinical information integration architecture which simulates lung cancer patients under treatment, a simulation based on IoT and pacemaker data, and an intelligent context-aware system based on digital twin framework. The reviewers conclude that digital twins have the power to transform the existing healthcare paradigm. Digital twins may accelerate digital transformation by enabling new forms of system coordination, predictive insight, and personalised care pathways.

#### AI

There were five reviews which covered AI applications in healthcare [44, 46, 48, 56, 58]. These covered deep learning applications for IoT in healthcare [56], AI and digital health for improving primary care services in Low and Middle Income Countries (LMICs) [48], integration into digital health systems [46, 58] and biotelemetry [58], and a review of a framework for interpretable ML systems in healthcare [44].

A notable emerging application of AI within healthcare is Large Language Models (LLMs). LLMs utilise large neural networks in order to mine unlabelled text data using a self-supervised learning approach [65]. They are then able to perform complex language related tasks such as text synthesis, translation, summarising, question-answering, etc. Since they are trained on large corpuses of text, they have significant potential in healthcare. Nasarian et al. (2024) discussed LLMs in their review into Interpretable ML (IML) systems and trust in healthcare [44]. Applications of LLMs in healthcare include chatbots (e.g., mental health), aiding in clinical documentation, data analysis, decision support, and translating medical data into patient friendly language. The authors raise that regulation of LLMs in healthcare poses a challenge due to safety, ethical standards, privacy. They also point out that without adequate human oversight, there is the potential for errors, misinformation, and biases. Enhanced interpretability is needed to integrate them in healthcare settings. They cite papers which have used Explainable AI (XAI) techniques to increase transparency within the model in the context of Mental Health Care, Medical Image Diagnosis, and Heart disease.

#### Big data

There were seven reviews which covered Big Data applications in healthcare [37, 42, 43, 47, 49, 51, 59]. Big data was reviewed in the context of exploring applications, challenges and future perspectives of Industry 4.0 [43], and design principles [51]. Big data analytics was reviewed in the context of IoT [47], decision support tools for cardiovascular care [59] and healthcare management [37], semantic web in healthcare [42], and alongside data science or ML [49].

Data fusing is an application of big data that holds significant potential in healthcare. Data fusing refers to the process of bringing together data from heterogenous datasets to extract a higher level of information from which to improve decision making. In healthcare, this could refer to big data extracted from IoT sensors and devices and wearables, then applying machine learning. Karatas et al. (2022) discussed this concept in their review [43]. The authors discuss the fusing of mobile application software technology, big data, sensing technology, and ML for Traditional Chinese Medicine (TCM)-oriented monitoring and assessment of healthcare. The system incorporated sensing, monitoring, time-series and image analysis, and a lifestyle app. This system was applied towards reducing the spread risk of COVID-19 during the pandemic and demonstrated a practical example of a health service using big data combined with data fusing.

#### IoT

There were five reviews which covered IoT applications in healthcare [40, 45, 47, 53, 56]. The reviews covered a survey of applications, challenges and future directions of IoT in healthcare [45], deep learning applications for IoT in health care [56], IoT-based healthcare systems in rural societies and governments [40], challenges and potential solutions [47] and mapping of research trends of IoT and data analytics [53].

Integration of personal monitoring devices and IoT allows for continuous remote monitoring of patient health. Heartbeat, body temperature, body mass, step count, and sleep quality all constitute health information routinely collected through wearable devices. Smart sensors and wearable devices provide the direct advantage of monitoring health status of individual patients, which can increase early diagnosis, prevent risk of serious illness, be used to manage chronic conditions, and contribute to personalised healthcare plans. Smart and wearable technologies can also provide big health data. Karatas et al. (2022) [43] review smart and wearable technologies in this context. They review a study which implemented AI techniques including Support Vector Machines (SVMs) and Long Short Term Memory (LSTM) algorithms, towards developing a Decision Support System (DSS). This can reduce cost and effort required for decision making by providing more accurate health data in the form of individualised risk maps. Studies where researchers used health monitoring via IoT combined with a text-mining ML algorithm to predict disease, and a wireless early prediction system for cardiac arrest were also reviewed, along with those studying use of Radio Frequency Identification (RFID), Wireless Sensor Network (WSN), and smart mobile devices to collect and make patient data accessible whilst within smart hospitals.

#### Blockchain

Two reviews covered Blockchain technology [39, 41]. Fatoum et al. (2021) reviewed integration of blockchain and digital technology in healthcare ecosystems [39]. Hameed et al. (2024) reviewed the integration of technologies, including blockchain, into healthcare systems, for sustainable development goals towards improving health and wellbeing [41].

Hameed et al. (2024) discuss the potential benefits and use cases of incorporating blockchain technologies into healthcare systems, comparing both the positives and negatives [41]. They characterise Blockchain technology by key factors: decentralization, immutability, security, privacy, consensus mechanism, anonymity, open source, smart contracts, and transparency. Authors list use cases of blockchain which include management of electronic health records (EHR), medical bill/Insurance claims, clinical trials and data, remote analysis/monitoring of patients, and analysis of health data. Fatoum et al. (2021) also cited EHR, patient monitoring, patient-centred interoperability, and clinical trials as major uses for blockchain technology [39].

**Table 5:** Inductive technology groups and their reported technologies, applications, settings, maturity levels, and socio-technical considerations

	Technologies reported	Application & focus	Healthcare settings	Maturity	Socio-technical considerations (SEIPS 2.0)	References
Integrated Technology / Industry 4.0	Additive manufacturing; 5G; artificial intelligence (AI)/ machine learning (ML); autonomous/adaptive robots; Big Data; biosensors; blockchain; digital twin (DT); augmented reality (AR)/virtual reality (VR); cloud computing; IoT; embedded systems; gamification; natural language processing (NLP); communication & networking; smart devices; mobile devices.	Clinical decision support systems (CDSS); health monitoring; telemedicine; targeted communication; digital apps; digital health platforms; cybersecurity; intelligent healthcare systems; mobile health; digital care optimisation.	NHS contexts; hospital systems; non-hospital long-term care; general healthcare services.	Mostly emerging (e.g., additive manufacturing), with many still experimental (e.g., DT, and robotics with AR/VR/AI for surgery). Some embedded (digital/mobile apps, CDSS, intelligent healthcare systems, AI/ML and AR/VR for rehab). Some disagreement between reviews for maturity.	People: Requires new digital skills; cross-disciplinary workflows; digital exclusion; altered patient-provider relationship. Tasks: Beginning automation of routine processes; introduction of cyber-physical workflows. Organisation: Strong dependency on interoperability, procurement redesign, and governance; changing requirements for system stability. Environment: Heavy reliance on infrastructure (5G, cloud, networking), safety (e.g., home-based rehabilitation).	[33–35, 38, 41, 50–52, 54, 55, 57, 60]
Artificial Intelligence (AI)	Physiological sensors; deep learning; ML; LLMs; CDSS; wearable health trackers; mHealth; telemedicine; AI– Internet of Things (IoT) fusion systems.	CDSS; patient monitoring; human activity recognition; enhanced trust and interpretability; virtual wards; data fusion; e-health optimisation; digital care twins (DCTs).	Primary healthcare services; healthcare systems; community care; general clinical workflows.	Emerging overall; some embedded (CDSS); LLMs still experimental. Numerous experimental studies on AI integration with biotelemetry devices.	People: Redistribution of cognitive tasks; concerns about trust, oversight, autonomy. Tasks: AI alters diagnostic and monitoring workflows; transparency concerns. Organisation: Requires validation, governance, regulation, safety structures. Environment: Data quality, integration, and documentation constraints.	[44, 46, 48, 56, 58]
Big Data	AI Adaptive robotics; additive manufacturing; Big Data; cloud computing; communication and networking; data analytics/fusion/mining; embedded systems; fog computing; IoT; ML; mobile apps; smart sensors; wearables; virtualization technologies.	Clinical decision support systems (electronic health records, EHRs; semantic web); health informatics; telematics; medical information exchange; cybersecurity; data privacy and security.	Healthcare organisations; health informatics departments; hospital systems.	Mostly embedded (EHR, informatics); analytics capabilities and integration with other advanced technologies (e.g., AI/NLP) emerging (fusion/mining).	People: Need for analytical literacy; increased documentation burden. Tasks: Data-driven reasoning shifts decision pathways. Organisation: Requires strong data governance and integration. Environment: Constrained by legacy systems and interoperability gaps.	[37, 42, 43, 47, 49, 51, 59]
Internet of Things (IoT)	5G networks; AI/ML/DL; Big Data analytics/collection; cloud systems; data fusion IoT-based healthcare technologies; fog computing; machine-to-man (M2M) communication; near-field communication (NFC); radio frequency identification (RFID); sensors; smart services; wireless sensor networks.	Health monitoring; human activity recognition; intelligent networks; Internet of Health Things (IoHT); remote care delivery.	Rural healthcare; telehealth systems; community health contexts.	Emerging, with experimental trials in diverse contexts (monitoring, diagnosis, symptom detection). Embedded in emergency and inpatient settings. Fast growing-ecosystem.	People: Increased patient involvement; greater technical expectations for staff. Tasks: Frequent monitoring reshapes thresholds for escalation and triage. Organisation: Requires device management, cybersecurity, and maintenance structures. Environment: Connectivity limitations affect reliability.	[40, 45, 47, 53, 56]
**Blockchain**	Smart contracts; blockchain–IoT systems; participant-controlled data access.	Interoperability; clinical trial data management; EHR sharing; remote patient monitoring; secure health data exchange.	Data-sharing networks; distributed healthcare ecosystems.	Mostly experimental; emerging in selected environments, with embedded blockchain-based EHR in Estonia.	People: New consent models; potential to increase patients’ trust in medical providers, new technical knowledge requirements, less doctor-patient interaction. Tasks: Changes verification and documentation practices. Organisation: Unclear governance, high implementation barriers, cost. Environment: Dependent on robust digital infrastructure, sustainability barriers.	[39, 41]

AI, Artificial Intelligence; ML, Machine Learning; DT, Digital Twin; VR, Virtual Reality; AR, Augmented Reality; IoT, Internet of Things; NLP, Natural Language Programming; National Health Service, NHS; LLM, Large Language Model; DCT, Digital Care Twin; Electronic Health Records, EHR; DL, Deep Learning; M2M, Machine-to-Machine Communication; NFC, Near Field Communication; RFID, Radio Frequency Identification; IoHT, Internet of Health Things

### Best-fit framework synthesis: digital transformation of healthcare in the literature

#### Conceptualisation of digital transformation in health services

The digital transformation of healthcare was the explicit focus of two included reviews. One reviewed mental health services [57] and one reviewed sustainable health and well-being [41]. It is referenced directly in a further five [34, 36, 46, 55, 60]. Across reviews, it is used to refer to the integration of technologies within healthcare systems as a recently emerging term (appearing as a keyword in just 1.3% of digital technology-related literature studied in a bibliometric analysis across two decades [34]). There is clear institutional recognition for the support of digital transformation of health services, which is reflected by various references in reviews to national policies, initiatives, and agendas to increase technological uptake in healthcare systems in Spain [55], India [36], and the UK [46, 57].

#### Technology readiness and implementation maturity

The current extent of digital transformation differs from country to country. Białczyk et al. (2024) defines e-health as e-prescriptions, electronic sick notes, electronic referrals, electronic medical records, mobile devices for health protection and health monitoring, internet patient accounts, remote medical care and electronic consultations, health mobile applications and health IT systems [55]. They state that the rate of e-health implementation is high in Northern Europe, but lower in Central and Eastern Europe. The implementation of more mature e-health technologies such as telemedicine and digital health records was more widespread and seen as a preparatory step towards implementing more advanced technologies such as AI. Bond et al. (2023) reviews integration of digital technologies into mental health services in the UK [57]. The authors determine three key uses for digital technologies in mental health service context to be optimising services, generating data to inform service-providers decisions, and providing new digital interventions. They discuss a suite of digital mental health interventions which ranges from mature technology such as digital health apps (e.g., mindfulness, mood tracking, patient management etc.), to more advanced technologies such as virtual reality (exposure therapy), natural language processing (semi-automated digital counselling), artificial intelligence (process mining), supervised machine learning (triaging and predicting outcomes), and robotics and sensors.

#### Reported outcomes of digital transformation

There were four reviews which reported outcomes with quantitative data. Two studies covered the effects of decision-support tools on quality of care [33, 59], one covered the use of digital technology to deliver healthcare services and health inequalities outcomes in the UK [38], and one covered the use of artificial intelligence and digital health on service delivery outcomes in LMICs [48]. Fanelli et al. (2023) reviewed big data analysis for decision making for management of healthcare organisations. They did not provide quantitative data, but did conduct a review for quality of care and quality of service outcomes [37]. We review the Quality of Care, Health Equality, Service Delivery, and Service Efficiency Outcomes. The outcomes and findings are listed in Table 6.

**Table 6:** Findings from reviews reporting care, equity, and service outcomes of digital transformation

	Author, Year	Intervention / Research Question	Outcome	Findings
Quality of Care Outcomes	Agarwal, et al., 2021	What are the effects of mobile phone supported clinical support decision tools (CDSTs) on quality of care outcomes in primary healthcare settings?	Providers’ adherence to recommended practices, guidelines, or protocols	Uncertain effect due to variations between studies or incomplete data. Two studies.
			Time between presentation and appropriate management	No studies.
			Patients’ or clients’ health behaviour	Little or no difference for reducing number of smokers Probably increases Aspirin adherence for those at cardiovascular disease (CVD) risk Little or no difference for increasing Diabetes medication adherence
			Patients’ or clients’ health status and well-being	Little or no difference to systolic blood pressure level among high risk CVD or to the number of women giving birth in a hospital. Little or no difference to HbA1c levels among people with poorly controlled diabetes, or to the number of people with hyperlipidaemia reaching LDL-cholesterol goals. Uncertain effect on maternal death, neonatal deaths, other maternal health outcomes
			Patients’ or clients’ acceptability and satisfaction	May improve satisfaction with the clarity or helpfulness of medication information among people with poorly controlled diabetes.
			Provider acceptability and satisfaction	No studies.
	Buzancic, et al., 2024	What are the effects on quality of care outcomes when using clinical support decision tools (CDSTs) for prevention of cardiovascular disease?	Quality of care outcomes (e.g., changes in prescribing, biomarkers, clinical target monitoring, completion of preventative care services)	A trend towards improvement in quality of care with the integration of CDSTs for blood pressure target attainment but not lipid and glucose clinical targets. Mixed results for screening/clinical tests completed/ordered.
			Patient-related outcomes (e.g., follow up on medication/appointment adherence, attainment of clinical biomarkers)	No significant difference in medication adherence between intervention and control groups. Statistically significant increase in interventions groups for appointment adherence.
	Fanelli et al., 2023	What are the effects of big data analysis regarding decision making for management of healthcare organisations (quality of care)?	Quality of care	Big data analytics can improve quality of care through improving predictive models and timing clinical activities, reducing waste by individualising treatments for greater impact on care pathway users’ behavioural styles, and for preventing spread of diseases, providing more effective care treatments, and improving knowledge transfer.
Health Equality Outcomes	Farre, et al., 2023	What are the effects of digital health interventions targeted at service users (Targeted service user communication and Personal health tracking), and service providers (Healthcare provider decision support, Telemedicine interventions) on health equality outcomes?	Implementation outcomes (acceptability, appropriateness, and feasibility)	Interventions show promise in improving engagement and awareness across various domains but face significant challenges in implementation. Structural barriers and insufficient consideration of diverse patient experiences risk limiting their effectiveness and potentially increasing health inequalities.
			Effectiveness outcomes (quality of life, intention to engage with marginalised groups, proportion of disadvantaged men binge drinking)	Digital health interventions can enhance psychological therapy for individuals with intellectual disabilities, support professional education to address marginalized groups, and aid in managing long-term conditions. Interventions remain better aligned with the needs of healthcare providers than those of service users from more disadvantaged populations.
Service Delivery Outcomes	Saif-Ur-Rahman, et al., 2023	What are the effects of artificial intelligence (AI) and digital health on primary care service delivery in Low and Middle Income Countries (LMICs)?	Service delivery outcomes (Use / recommendation of m-health, Record-keeping and documentation, Waiting time, Efficiency of services, Client satisfaction, and Quality of service)	Use of m-health improved record-keeping, shortened waiting time, competency of health workers, clinical attendance, follow up, medication adherence, knowledge (but not reduction in risk of disease), interaction, information sharing, and clinic attendance. Reported data includes a decrease in treatment time from 4 to 3 days, that 11% of patients preferred face-to-face monitoring, clients’ satisfaction regarding health workers’ competencies rose from 11% to 89%, and that 93% reported that m-health apps helped them emotionally. They conclude that m-health shortened waiting times for clients and service providers, and minimised crowding at facilities.
Service Efficiency Outcomes	Fanelli, et al., 2023	What are the effects of big data analysis regarding decision making for management of healthcare organisations (quality of service)?	Quality of service	Big data analytics can reduce costs and improve business planning and patient experience and satisfaction. Identified two main themes: the use of big data to generate greater value for the healthcare organisation and patient, and the accessibility of health services. They group the main aspects of big data analysis which increases healthcare service quality into five main areas: traceability, analysis, speed of decision-making, forecasting and interoperability.

CDST, Clinical Support Decision Tool; CVD, Cardiovascular Disease; HbA1c, Haemoglobin A1c; LDL, low-density lipoprotein; AI, Artificial Intelligence; LMICs, Low and Middle Income Countries

#### Digital transformation in the United Kingdom

There were five included reviews published in the UK or which discussed UK services [38, 46, 57, 64]. These discussed an intervention and classification framework for digital public health interventions [64], digital transformation of mental health services [57], digital technologies in healthcare service delivery and health inequalities [38] and digital health and care following the COVID-19 pandemic [46]. Key focus and findings are presented in Additional File 4 - UK Findings Table.docx.

#### Barriers and challenges of digital transformation

There were 11 reviews which discussed barriers and challenges related to the application of digital technologies within health systems [36–38, 41–43, 46, 50, 55, 57, 60]. The barriers were Usability and Accessibility [42, 57], Care Quality and Evidence [46, 50, 57, 60], Cost [55], Data and Trust [37, 41, 43, 55, 57], Ethics and Equity [37, 38, 41, 60], Implementation Challenges [36, 41, 46], and Threats to Professional’s Authority [60]. Reviews referencing digital transformation directly cite two main negative impacts of the digital transformation of healthcare, which were barriers to Implementation and Overuse of technology. Barriers to implementation include coordination of integration whilst maintaining equity and value [60], requirements for technical knowledge, and privacy of patients information [41], and challenges in ethics and adoption [57]. Concerns regarding overuse of technology include the need to maintain vigilance against viewing technology as a silver bullet to solve long-standing issues [46] and digital solutionism [57], overpromotion of screen time [57], reduced doctor–patient interaction, over-reliance on technology, and a resultant increase in social imbalance [41].

## Discussion

There is a wide conceptual scope for what constitutes the digital transformation of healthcare, encompassing a range of contexts, technology types, and applications. In this systematic scoping review and best-fit framework synthesis, we synthesised evidence across 28 reviews reporting on 1606 individual studies (with an additional 2437 included in a bibliometric analysis), to produce a broad overview of state-of-the-art technologies used in the design and operation of healthcare systems. This paper offers an analytical synthesis that highlights the maturity, application, and outcomes of technologies implemented within health services to support digital transformation.

### Key technologies and maturity

Across the screened literature, the three most frequently reported general technology terms were digital health, digital technologies, and telemedicine. These terms often overlapped in their use. “Digital health” typically referred to digital platforms such as mobile health apps, which fall within the definition of digitisation, while “digital technologies” encompassed both mature digitisation/digitalisation technologies (e.g., e-health, telemedicine) and more advanced innovations like AI, IoT, and Big Data analytics. The frequent appearance of these topics in the literature reflects their widespread implementation and maturity, particularly in response to increased demand for remote service delivery during the COVID-19 pandemic [35].

With a focus on state-of-the-art technologies, five primary technology groups emerged from our systematic review: Integrated Technology/Industry 4.0, Artificial Intelligence, Big Data, Internet of Things, and Blockchain. We mapped these technologies to a bespoke functional technology framework whereby the groups corresponded to data capture (IoT), security and validation (blockchain), data aggregation (big data), data analytics (AI), and technologies for system-level implementation and coordination (industry 4.0), respectively. Technologies were applied in diverse settings including primary care, long-term care, rural healthcare, mental health services, and national healthcare systems such as the NHS. Integrated technology, often framed within the Industry 4.0 paradigm, was the most represented group. It incorporated elements such as smart devices, cloud computing, and data-driven platforms used for clinical decision-making, diagnostics, remote monitoring, and care optimisation. However, as many Industry 4.0 technologies are still emerging [52], robust real-world studies are needed to determine their effectiveness, scalability, and integration into routine clinical practice.

Of particular relevance to the current era are advanced systems like AI and IoT, which enable automation, real-time decision support, and predictive analytics. For instance, LLMs, a form of deep learning, have shown potential in supporting clinical documentation, chatbots for mental health, and patient-facing data translation. However, their interpretability and regulation remain challenging​ and evidence is limited since they have not been integrated into real-world clinical workflows at scale. Similarly, IoT-enabled smart sensors and wearables were discussed as key for personalised care and chronic disease management. The potential for feeding data from sensors and wearables into heterogenous patient-centred models using processes such as data fusing stands to improve clinical decision making and provide timely response. However, challenges with implementation remain, including data security, integration into clinical workflows, device designs, and reliability [45]. There may also be challenges in achieving cross-platform equivalence and standardisation across clinics to ensure consistent performance.

While this review identifies a wide range of digital technologies applied across healthcare contexts, the included reviews tended to focus on identifying technologies, with few distinguishing between technology deployment and digital transformation. Many reviews, by design, either listed tools or described the adoption of specific tools without examining whether these produced broader changes in care models, workflows, or workforce roles. This reflects a bias within our sample towards digitalisation over conceptual awareness of digital transformation.

### National and regional progression of the digital transformation

The implementation of technologies for the digital transformation of healthcare varies significantly in rate and the sophistication of technologies across regions. Białczyk et al. (2024) reported that Northern European countries have achieved substantial progress in adopting mature technologies such as e-prescriptions and digital records, whereas Central and Eastern Europe lag behind in adoption rates [55]​. Considering the fact that the biggest challenges were identified as financing innovation and improving digital skills [55], regional variation in the pace of digital transformation likely reflect differences in policy and capacity related to long-term investment, interoperability standards, and digital workforce capacity.

Within the UK, digital health interventions are increasingly embedded within the NHS, particularly in mental health services, where Bond et al. (2023) identified the use of digital apps, chatbots, and AI tools, but also highlighted serious concerns around clinical assurance and data privacy [57]​. Additionally, challenges related to digital inclusion persist [38]. Many populations with the highest health needs, such as older adults, individuals with low digital literacy, or those lacking internet access, are often least well served by digital-first approaches [38]. Failure to consider the needs of these diverse populations may inhibit the potential benefit of technology implementation. It was also reported that interventions may be better aligned with the needs of providers than those of service users from more disadvantaged populations [38], which underscores the need for sufficient study within all relevant stakeholder groups, as well as the practical considerations. These contradictions highlight the need for digital transformation strategies that move beyond technological capability to address the organisational, regulatory, and social foundations required for equitable, sustainable system change.

### Outcomes of implementation

We assessed four reviews which provided quantitative evidence on the impact of digital technologies in health services. The reviews covered a range of outcomes covering four main domains: quality of care, health equality, service delivery, service and efficiency. The analysis provides examples of assessments undertaken for the implementation of technologies within healthcare, and which stakeholder groups they were important for.

Quality of care outcomes were typically relevant to patient populations managed by healthcare staff and were measured using healthcare indicators such as clinical biomarkers, as well as service metrics including the number of screening or clinical tests completed, time to appointment, and practitioners’ adherence to guidelines or protocols. A study on CDSTs [33] identified studies which recorded patient behaviour, health and satisfaction reports, but did not find any studies reporting provider acceptability metrics. This could present problems if staff are unprepared to deal with alterations to their workflow or do not have requisite skills.

Health equity outcomes showed the potential of digital interventions to enhance access and engagement [38], and were measured through implementation and effectiveness outcomes. However, significant barriers remain, particularly for disadvantaged groups such as those explored above (older adults, individuals with low digital literacy, and those lacking internet access), where technology risks exacerbating existing inequalities if needs of these populations are not properly addressed.

Service delivery outcomes were mainly relevant to healthcare workers and patients, with AI and mobile health tools improving record-keeping, waiting times, and satisfaction in LMICs​ [48]. Whilst successful implementation of AI into clinical workflows has been demonstrated in primary care in high-income countries [66], large scale multi-setting study designs are required to better understand implications in LMICs for primary healthcare systems.

Service efficiency outcomes held greatest significance for healthcare managers. Big data tools were reported to improve better planning, forecasting, and personalisation of care​ [37], though technical and cultural training will be required for healthcare professionals to gain maximum benefit for informing decision-making processes.

Notably absent within our review were outcomes related to continuity of care, care pathway redesign, or workforce transformation, which are core to the idea of digital transformation as organisational change. Whilst this review identified a range of outcome measures for different aspects of implementation, this paper was not conceived as a comprehensive review for determining the efficacy of implementation for any specific technology. Future systematic reviews or meta-analyses should assess the organisational effects of specific implementation cases of more targeted technology and constrained scope.

### Barriers and challenges

Despite broad enthusiasm for the adoption of technology for the transformation of healthcare services, this review identified multiple barriers to successful implementation. These include concerns about usability and accessibility, the quality of supporting evidence, costs, data privacy and trust, equity and ethical concerns, and implementation logistics.

The cost barriers for technology implementation [55] are ubiquitous, but are very much a context-dependent issue. Policy dictating healthcare structure, financial constraints, and workforce culture and capacity are strong deterministic factors influencing institutional inertia. Whilst in principle (with the appropriate technology and use) the digital transformation aims to improve cost saving and efficiency, there is also a cost associated with ensuring that it is correctly implemented. Detailed studies into capacity difference between institutions are therefore required to ensure that technology applications are not restricted to more affluent systems (or segments of systems) [60].

There is also evidence of resistance from professionals concerned about authority and control, especially in digitally enabled care models​​​ [60]. These threaten the power dynamics within clinical practice by having the potential to reduce perceived value or trust placed in physicians. Physicians are also wary of barriers the lack of transparency of LLMs and the potential for overreliance and digital solutionism technologies such as mental health apps pose to adoption [57], while patients might lack trust in AI diagnostics [67]. Clinical trials are on-going to ensure the feasibility and validity of big data in clinical applications.

Challenges related to patient-facing technology implementation are frequently compounded by persistent digital exclusion, raising concerns that digital-first approaches may exacerbate existing health inequalities if implementation is insufficiently attuned to user needs [38]. This has implications for contested values within healthcare regarding equity, access, and responsibility. Digital-first approaches may prioritise system efficiency and remote delivery, while disadvantaging individuals with limited digital literacy or access to technology. When implementation does not adequately account for these needs, digital interventions risk exacerbating existing health inequalities [36], raising questions about whose needs are being prioritised in digitally enabled models of care.

While many technologies have been implemented in health services, the evidence base for more advanced, integrated technologies such as real-time Digital Twins or cross-platform AI-IoT systems, is notably limited. Few reviews examined these within real-world settings at scale, particularly regarding long-term impacts, integration challenges, and ethical implications.

Taken together, these findings indicate a need for future research to shift from technology-centred evaluations towards socio-technical and organisational studies that examine how digital systems are embedded within care pathways, across professional and patient groups, and governed at institutional level. Longitudinal, real-world studies that integrate outcomes for patients, professionals, and health systems will be critical for determining how new technologies can be integrated as part of a broader digital transformation effort.

## Conclusions

This synthesis highlights several implications for multiple stakeholder groups within the healthcare ecosystem. For policymakers and health system executives, the synthesis identifies technologies that have been shown to improve system efficiency and integration. Service managers and clinicians can use this evidence to align selection of digital tools with clinical workflows and service redesign. With the evidence we present, stakeholders occupying these positions within the UK might be better able to make informed decisions regarding implementation of technologies within the context of the ongoing transformation of the NHS. Mature technologies like telemedicine and mobile health platforms can serve as the foundation for broader digital transformation efforts. Future implementations should be designed with equity in mind, ensuring that digital solutions are accessible and inclusive. More rigorous evaluations are needed for advanced technologies such as LLMs, Digital Twins, and AI-driven systems to understand their impact on care quality and system efficiency. Future research is required to evaluate the effectiveness and scalability of digital technologies at the system and service levels, specifically into real-world implementation and outcomes. Considering the diversity in settings across the globe, comparative studies will also be required to identify context-specific challenges. Finally, national strategies, such as those in the NHS, must include robust frameworks for ethical oversight, clinical assurance, and user-centred design to maximise benefit while minimising harm.

### Study limitations

A key limitation of this study is the heterogeneity of technologies, settings, and outcomes across included reviews. The global variation in adoption and implementation strategies makes it difficult to generalise findings, and the fast pace of technological advancement means that even recent reviews may quickly become outdated. There may have been some overlap in the primary literature across the systematic reviews included. We chose not to examine this in depth since it is of little consequence to the main aim of this review, which was the identification of these technologies and the context around their implementation. Furthermore, when drawing from international literature, language requirements may limit the publications which it is possible to include. We also encountered considerable variability in terms of the methodological quality of reviews.

## Electronic supplementary material

Below is the link to the electronic supplementary material.

Supplementary Material 1

Supplementary Material 2

Supplementary Material 3

Supplementary Material 4

## Data Availability

All data generated or analysed during this study are included in this published article and its supplementary information files.

## Abbreviations

AI: Artificial Intelligence
AMED: The Allied and Complementary Medicine Database
AR: Augmented reality
CDAS: Child Development & Adolescent Studies
CDST: Clinical Support Decision Tool
CINAHL: Cumulative Index to Nursing and Allied Health Literature
CNN: Convolutional neural network
CVD: Cardiovascular Disease
DCHC: Data-Centric Health-Care
DCT: Digital Care Twin
DL: Deep Learning
DR: Digital Rehabilitation
DSS: Decision Support System
DT: Digital twin
EHR: Electronic Health Records
HbA1c: Haemoglobin A1c
HMIC: Health Management Information Consortium
HMIS: Health Management Information System
IML: Interpretable Machine Learning
IoHT: Internet of Health Things
IoT: Internet of Things
JBI: Joanna Briggs Institute
LDL: Low-density lipoprotein
LLM: Large Language Model
LMICs: Low and Middle Income Countries
LSTM: Long Short Term Memory
M2M: Machine-to-Machine Communication
MetaQAT: Meta Quality Appraisal Tool
ML: Machine Learning
MMAT: Mixed-Methods Appraisal Tool
NFC: Near Field Communication
NHS: National Health Service
NICE: National Institute for Health and Care Excellence
NLP: Natural Language Programming
NS: Not Specified
ORCHA: Organisation for the Review of Care and Health Apps
PICO: Participant, Intervention, Comparator, Outcome
PICo: Population, Phenomena of interest, Context
PRISMA: Preferred Reporting Items for Systematic Reviews and Meta- Analyses
RFID: Radio Frequency Identification
RoB: Risk of Bias
SDG: Sustainable development goal
SPP: Social Policy and Practice
SVM: Support Vector Machine
TCM: Traditional Chinese Medicine
VR: Virtual Reality
WSN: Wireless Sensor Network
XAI: Explainable Artificial Intelligence
