# Supplementary material for: State-of-the-art technologies for the digital transformation of healthcare services – a systematic scoping review
Source: BMC Health Serv Res. 2026 Jun 27;26:877. doi: 10.1186/s12913-026-14714-9 (PMC13312714; doi:10.1186/s12913-026-14714-9)
Supplement: Supplementary file 1 — Supplementary Material 1 [file 12913_2026_14714_MOESM1_ESM.docx]

## **Search Strategy and Results for “State-of-the-art technologies for the digital transformation of healthcare services – a systematic review and framework synthesis of reviews”**

## **Search strategy**

We used the PICo (Population, Phenomena of interest, Context) framework following the Joanna Briggs Institute (JBI) methodology [1]. This framework, often applied to mixed-methods reviews, was better suited to our research goals than the often used PICO (Participant, Intervention, Comparator, Outcome) framework [1], since no restrictions were applied to “Outcome”, and “Comparator” for studies measuring effects was “usual clinical practice” or equivalent. **Table 1** shows the three components of the search strategy: “(Re)design of Health Systems/Services” (Population), “Digital Transformation/Digital Technology” (Phenomenon of Interest), and “Study Design” (Context). The “Digital Transformation/Digital Technology” component included terms related to digital technologies, and specific technologies of interest, and the “Study Design” aspect included synonyms for reviews and review types of interest.

**Supplementary Table 1. PICo items.**

| **P** | Population | Healthcare Systems/Service Design/Redesign |
| --- | --- | --- |
| **I** | Phenomenon of interest | Digital Transformation/Digital Technology |
| **Co** | Context | Study Design |

*P, Population; I, Phenomena of Interest, Co, Context.*

For Phenomena of Interest, a summary of the search terms is included in **Table 2**.

**Supplementary Table 2. Summary of terms for Phenomena of Interest.**

| Digital Transformation |
| --- |
| Digital |
| Digitalisation |
| Digitisation |
| Digital Shadow |
| Digital Twin |
| Symbiotic Simulation |
| Realtime Simulation |
| Big Data |
| Realtime Data |
| Cloud Computing Data |
| Sensor Data |
| (Industrial) Internet of Things (IoT) |

*IoT, Internet of Things.*

During preliminary scoping, we also developed searches, including relevant synonyms and methods, for artificial intelligence (AI) / machine learning (ML), and for computer modelling and simulation for inclusion within the Phenomena of interest term. However, these returned large numbers of hits since they are large topics within their own right and will therefore be explored separately. **Table 3** and **Table 4** show results for AI/ML and computer modelling/simulations, respectively. Results are from OVID Medline, one of 11 databases explored.

**Supplementary Table 3. Search results for artificial intelligence / machine learning from Ovid Medline.**

|  | **AI/ML** |  |
| --- | --- | --- |
| 1 | Machine Learn*.ti,ab,sh. | 116456 |
| 2 | Supervised learn*.ti,ab,sh. | 6130 |
| 3 | Unsupervised Learn*.ti,ab,sh. | 2578 |
| 4 | Reinforcement Learn*.ti,ab,sh. | 6718 |
| 5 | deep learn*.ti,ab,sh. | 63682 |
| 6 | (Artificial Intelligence or AI).ti,ab,sh. | 109100 |
| 7 | or/1-6 | 265134 |

**Supplementary Table 4. Search results for computer modelling and simulation from Ovid Medline.**

|  | **Computer Modelling and Simulation**[2] |  |
| --- | --- | --- |
| 1 | exp Computer Simulation/ | 314351 |
| 2 | models, statistical/ | 100864 |
| 3 | ((computer* or distributed or hybrid) adj6 (model* or simulation*)).ti,ab,sh. | 212596 |
| 4 | ((flow or simulation) adj6 model$).ti,ab,sh. | 67192 |
| 5 | (system adj3 model$).ti,ab,sh. | 80058 |
| 6 | (discrete adj2 event).ti,ab,sh. | 1706 |
| 7 | (monte adj2 carlo).ti,ab,sh. | 62625 |
| 8 | markov.ti,ab,sh. | 30193 |
| 9 | (statechart$ or stochastic or system dynamics).ti,ab,sh. | 56350 |
| 10 | ("SD" adj1 (model* or simul*)).ti,ab,sh. | 488 |
| 11 | (agent adj2 based).ti,ab,sh. | 8817 |
| 12 | or/1-11 | 687185 |

Search terms were adapted from existing reviews on topics related to “(Re)design [3] of Health Systems [4] /Services [3]”, “Digital Transformation [5]”, as well as other terms added manually by the authors. For “Study Design”, we adapted existing search filters for reviews [6]. The search strategy was first developed for Ovid Medline, then translated for other platforms where necessary, using the Polyglot tool developed at Bond University [8]. Appropriate subject headings were selected for each search term for databases that used them. The number of results for the subject headings were then checked across and between databases to confirm, and the results retrieved.

## **Results**

**Supplementary Table 5. Final number of results across databases.**

|  | **Ovid** | **EBSCO** | **SCOPUS** | **WoS** | **Total** | **Total (unique)** |
| --- | --- | --- | --- | --- | --- | --- |
| **Number of results** | 629 | 115 | 493 | 186 | 1423 | 1011 |

### **OVID**

Search date: 4th November 2024

Embase <1974 to 2024 November 01>

HMIC Health Management Information Consortium <1979 to September 2024>

Ovid MEDLINE(R) ALL <1946 to November 01, 2024>

APA PsycInfo <1806 to October 2024 Week 4>

Social Policy and Practice <202410>

**Supplementary Table 6. OVID search strategy and results.**

|  | **(Re)design of Health Systems/Services (P)** |  |
| --- | --- | --- |
| 1 | ((design* or redesign*) adj10 (health or healthcare or "health care" or health*) adj10 (service or system or services or systems or system-level or systems-level)).ti,ab. | 37680 |
| 2 | limit 1 to yr="2019 - 2024" | 14725 |
| 3 | health service/ or health services/ or Health Services/ or Health Care Services/ | 278700 |
| 4 | limit 3 to yr="2019 - 2024" | 48250 |
| 5 | 2 or 4 | 62039 |
|  | **Digital Transformation/Digital Technology (I)** |  |
| 41 | digital transformation.ti,ab. | 3453 |
| 42 | digital*.ti,ab. | 547912 |
| 43 | digitali?ation.ti,ab. | 8710 |
| 44 | digiti*.ti,ab. | 50891 |
| 45 | digiti?ation.ti,ab. | 8415 |
| 46 | or/41-45 | 587814 |
| 47 | digital shadow.ti,ab. | 17 |
| 48 | digital twin.ti,ab. | 1897 |
| 49 | symbiotic simul*.ti,ab. | 7 |
| 50 | (real-time simul* or realtime simul*).ti,ab. | 1370 |
| 51 | (RtS and (simul* or model*)).ti,ab. | 5550 |
| 52 | or/47-51 | 8814 |
| 53 | Big data.ti,ab. | 33394 |
| 54 | (real-time data or realtime data).ti,ab. | 7477 |
| 55 | (Cloud comput* adj3 data).ti,ab. | 741 |
| 56 | sensor data.ti,ab. | 8613 |
| 57 | IIOT.ti,ab. | 493 |
| 58 | (industrial and (IOT or Internet of Things)).ti,ab. | 1539 |
| 59 | or/53-58 | 50812 |
| 60 | 46 or 52 or 59 | 640858 |
| 61 | limit 60 to yr="2019 - 2024" | 288326 |
| 62 | digital technology/ or Digital Technology/ | 11012 |
| 63 | limit 62 to yr="2019 - 2024" | 10679 |
| 64 | 61 or 63 | 290751 |
|  | **Reviews (C)** |  |
| 97 | review*.ab,ti. or review.pt. or overview*.ab,ti. or meta-analy*.ab,ti. or metaanaly*.ab,ti. or metanaly*.ab,ti. or meta-analysis.pt. or Meta-Analysis/ or meta-regression*.ab,ti. or metaregression*.ab,ti. or (meta adj regression*).ab,ti. or search*.ab. or synthes*.ab,ti. or metasynthes*.ab,ti. or meta-synthes*.ab,ti. or metaethnograph*.ab,ti. or meta-ethnograph*.ab,ti. | 14577856 |
| 98 | 5 and 40 and 96 | 234 |
| 99 | 5 and 40 and 97 | 940 |
| 100 | 5 and 64 and 96 | 249 |
| 101 | 5 and 64 and 97 | 770 |
|  | **Health System/Service + Digital Transformation + Reviews (P+I+C)** |  |
| 105 | remove duplicates from 101 | 629 |

### **EBSCO**

AgeLine, AMED - The Allied and Complementary Medicine Database, Child Development & Adolescent Studies, CINAHL Ultimate

Search date: 4th November 2024

**Supplementary Table 7. EBSCO search strategy and results.**

|  | **(Re)design of Health Systems/Services (P)** |  |
| --- | --- | --- |
| S1 | (((TI design* OR AB design*) OR (TI redesign* OR AB redesign*)) N10 ((TI health OR AB health) OR (TI healthcare OR AB healthcare) OR (TI "health care" OR AB "health care") OR (TI health* OR AB health*)) N10 ((TI service OR AB service) OR (TI system OR AB system) OR (TI services OR AB services) OR (TI systems OR AB systems) OR (TI system-level OR AB system-level) OR (TI systems-level OR AB systems-level)))  Limiters - Publication Date: 20190101-20241231 | 3,417 |
|  | **Digital Transformation/Digital Technology (I)** |  |
| S28 | (TI "digital transformation" OR AB "digital transformation") | 376 |
| S29 | (TI digital* OR AB digital*) | 62,696 |
| S30 | (TI digitali#ation OR AB digitali#ation) | 768 |
| S31 | (TI digiti* OR AB digiti*) | 4,231 |
| S32 | (TI digiti#ation OR AB digiti#ation) | 888 |
| S33 | S28 OR S29 OR S30 OR S31 OR S32  Limiters - Publication Date: 20190101-20241231 | 32,243 |
| S34 | (TI "digital shadow" OR AB "digital shadow") | 33 |
| S35 | (TI "digital twin" OR AB "digital twin") | 75 |
| S36 | (TI "symbiotic simul*" OR AB "symbiotic simul*") | 1 |
| S37 | ((TI "real-time simul*" OR AB "real-time simul*") OR (TI "realtime simul*" OR AB "realtime simul*")) | 54 |
| S38 | ((TI RtS OR AB RtS) AND ((TI simul* OR AB simul*) OR (TI model* OR AB model*))) | 450 |
| S39 | S34 OR S35 OR S36 OR S37 OR S38 | 580 |
| S40 | (TI "Big data" OR AB "Big data") | 3,496 |
| S41 | ((TI "real-time data" OR AB "real-time data") OR (TI "realtime data" OR AB "realtime data")) | 621 |
| S42 | ((TI "Cloud comput*" OR AB "Cloud comput*") N3 (TI data OR AB data)) | 62 |
| S43 | (TI "sensor data" OR AB "sensor data") | 500 |
| S44 | (TI IIOT OR AB IIOT) | 7 |
| S45 | ((TI industrial OR AB industrial) AND ((TI IOT OR AB IOT) OR (TI "Internet of Things" OR AB "Internet of Things"))) | 29 |
| S46 | S40 OR S41 OR S42 OR S43 OR S44 OR S45 | 4,654 |
| S47 | S33 OR S39 OR S46  Limiters - Publication Date: 20190101-20241231 | 34,699 |
|  | **Reviews (C)** |  |
| S58 | (AB(review* OR overview* OR meta-analy* OR metaanaly* OR metanaly* OR meta-regression* OR metaregression* OR (meta N1 regression*) OR search* OR synthes* OR metasynthes* OR meta-synthes* OR metaethnograph* OR meta-ethnograph*) OR TI(review* OR overview* OR meta-analy* OR metaanaly* OR metanaly* OR meta-regression* OR metaregression* OR (meta N1 regression*) OR synthes* OR metasynthes* OR meta-synthes* OR metaethnograph* OR meta-ethnograph*) OR PT("Systematic Review") OR PT("Meta Synthesis") OR PT("Meta Ethnography") OR MH("Meta Analysis")) NOT PT("Book Review") | 1,356,451 |
| S59 | (MH "Health Services") OR (MH "Health Systems")  Limiters - Publication Date: 20190101-20241231 | 10,021 |
| S60 | (MH "Systems Design") OR (MH "Systems Analysis") OR (MH "Systems Theory") OR (MH "Patient Centered Care")  Limiters - Publication Date: 20190101-20241231 | 15,558 |
| S61 | (MH "Digital Health") OR (MH "Digital Technology")  Limiters - Publication Date: 20190101-20241231 | 6,747 |
| S62 | S1 OR S59  Limiters - Publication Date: 20190101-20241231 | 13,240 |
| S63 | S27 OR S60  Limiters - Publication Date: 20190101-20241231 | 27,031 |
| S64 | S47 OR S61  Limiters - Publication Date: 20190101-20241231 | 36,973 |
|  | **Health System/Service + Digital Transformation + Reviews (P+I+C)** |  |
| S68 | S62 AND S64 AND S58 | 115 |

### **SCOPUS**

Search date: 4^th^ November 2024

**Supplementary Table 8. SCOPUS search strategy and results.**

|  | **(Re)design of Health Systems/Services (P)** |  |
| --- | --- | --- |
| 1 | ( TITLE-ABS ( ( design* OR redesign* ) W/10 ( health OR healthcare OR "health care" OR health* ) W/10 ( service OR system OR services OR systems OR system-level OR systems-level ) ) ) AND PUBYEAR > 2018 AND PUBYEAR < 2025 | 20,761 |
|  | **Digital Transformation/Digital Technology (I)** |  |
| 3 | ( TITLE-ABS ( "digital transformation" OR "digital*" OR "digitali*ation" OR "digiti*" OR "digiti*ation" OR "digital shadow" OR "digital twin" OR "symbiotic simul*" OR "real-time simul*" OR "realtime simul*" OR ( "RtS" AND ( "simul*" OR "model*" ) ) OR "Big data" OR "real-time data" OR "realtime data" OR "Cloud comput* data" OR "sensor data" OR "IIOT" OR ( "industrial" AND ( "IOT" OR "Internet of Things" ) ) ) ) AND PUBYEAR > 2018 AND PUBYEAR < 2025 | 684,579 |
|  | **Reviews (C)** |  |
| 5 | ( TITLE-ABS(review*) OR DOCTYPE(review) OR TITLE-ABS(overview*) OR TITLE-ABS(meta-analy*) OR TITLE-ABS(metaanaly*) OR TITLE-ABS(metanaly*) OR DOCTYPE(meta-analysis) OR INDEXTERMS(Meta-Analysis) OR TITLE-ABS(meta-regression*) OR TITLE-ABS(metaregression*) OR TITLE-ABS(meta W/1 regression*) OR ABS(search*) OR TITLE-ABS(synthes*) OR TITLE-ABS(metasynthes*) OR TITLE-ABS(meta-synthes*) OR TITLE-ABS(metaethnograph*) OR TITLE-ABS(meta-ethnograph*) ) AND PUBYEAR > 2018 AND PUBYEAR < 2025 | 3,469,473 |
|  | **Health System/Service + Digital Transformation + Reviews (P+I+C)** |  |
| 9 | 1 AND 3 AND 5 | 493 |

### **Web of Science**

Web of Science Core Collection.

Search date: 4th November 2024

**Supplementary Table 9. Web of Science search strategy and results.**

|  | **(Re)design of Health Systems/Services (P)** |  |
| --- | --- | --- |
| 1 | (TI=((design* OR redesign* ) NEAR/10 (health OR healthcare OR "health care" OR health* ) NEAR/10 (service OR system OR services OR systems OR system-level OR systems-level )) OR AB=((design* OR redesign* ) NEAR/10 (health OR healthcare OR "health care" OR health* ) NEAR/10 (service OR system OR services OR systems OR system-level OR systems-level )))  Last 5 years | 7,659 |
|  | **Digital Transformation/Digital Technology (I)** |  |
| 3 | TI= ( "digital transformation" OR "digital*" OR "digitali*ation" OR "digiti*" OR "digiti*ation" OR "digital shadow" OR "digital twin" OR "symbiotic simul*" OR "real-time simul*" OR "realtime simul*" OR ( "RtS" AND ( "simul*" OR "model*" ) ) OR "Big data" OR "real-time data" OR "realtime data" OR "Cloud comput* data" OR "sensor data" OR "IIOT" OR ( "industrial" AND ( "IOT" OR "Internet of Things" ) ) ) OR AB= ( "digital transformation" OR "digital*" OR "digitali*ation" OR "digiti*" OR "digiti*ation" OR "digital shadow" OR "digital twin" OR "symbiotic simul*" OR "real-time simul*" OR "realtime simul*" OR ( "RtS" AND ( "simul*" OR "model*" ) ) OR "Big data" OR "real-time data" OR "realtime data" OR "Cloud comput* data" OR "sensor data" OR "IIOT" OR ( "industrial" AND ( "IOT" OR "Internet of Things" ) ) )  Last 5 years | 375,495 |
|  | **Reviews (C)** |  |
| 5 | TS=(review* OR overview* OR meta-analy* OR metaanaly* OR metanaly* OR meta-analysis OR meta-regression* OR metaregression* OR search* OR synthes* OR metasynthes* OR meta-synthes* OR metaethnograph* OR meta-ethnograph*)  Last 5 years | 2,570,360 |
|  | **Health System/Service + Digital Transformation + Reviews (P+I+C)** |  |
| 13 | #1 AND #3 AND #5 | 186 |

## **References**

1. JBI Manual for Evidence Synthesis. JBI Manual for Evidence Synthesis. 2024. https://doi.org/10.46658/jbimes-24-01.

2. Mohiuddin S, Busby J, Savović J, Richards A, Northstone K, Hollingworth W, et al. Patient flow within UK emergency departments: A systematic review of the use of computer simulation modelling methods - Search Strategy. Europe’s Brexit. 2017;27:263–6.

3. Vaz N, Araujo CAS. Service design for the transformation of healthcare systems: A systematic review of literature. Health Services Management Research. 2023;37:174–88.

4. Mbau R, Musiega A, Nyawira L, Tsofa B, Mulwa A, Molyneux S, et al. Analysing the Efficiency of Health Systems: A Systematic Review of the Literature. Applied Health Economics and Health Policy. 2023;21:205–24.

5. Egodawele M, Sedera D, Bui V. A Systematic Review of Digital Transformation Literature (2013 – 2021) and the development of an overarching a-priori model to guide future research. ACIS 2022 Proceedings. 2022.

6. Melendez-Torres GJ, Dowdell EB, Kelley SJ, Lewis-O’Connor A. Strategies for Identification of Intimate Partner Violence in Healthcare Settings: Overview of Reviews and Realist Synthesis. Journal of Family Violence. 2023;38:1457–68.

7. CADTH. RCT / CCT - MEDLINE, Embase. Search Filters Database. 2024.

8. Clark JM, Sanders S, Carter M, Honeyman D, Cleo G, Auld Y, et al. Improving the translation of search strategies using the polyglot search translator: A randomized controlled trial. Journal of the Medical Library Association. 2020;108:195–207.
