# Supplementary material for: State-of-the-art technologies for the digital transformation of healthcare services – a systematic scoping review
Source: BMC Health Serv Res. 2026 Jun 27;26:877. doi: 10.1186/s12913-026-14714-9 (PMC13312714; doi:10.1186/s12913-026-14714-9)
Supplement: Supplementary file 4 — Supplementary Material 4 [file 12913_2026_14714_MOESM4_ESM.docx]

**Supplementary Table 10. Findings from reviews with focus on UK context.**

| **Author, Year** | **Review Aim / Focus** | **Relevance to UK Context** | **Key Findings** |
| --- | --- | --- | --- |
| Wienert et al., 2022 | Define classification framework for public digital health interventions. | Created technology classification framework by merging three existing ones, including one from UK’s National Institute for Health and Care Excellence (NICE). | Demonstrates that technology classification frameworks exist within UK, though focus is on digital public health interventions rather than technologies to be used for systems-level design. |
| Bond et al., 2023 | Integration of digital technologies for mental health services with focus on the UK. | Discusses a range of digital technologies and their applications within UK mental health services, such as chat bots that have recently been implemented within traditional NHS services. | Discusses ethical challenges and cites that only 32% of mental health apps would pass a quality assurance benchmarks for user experience, data privacy, and aspects of clinical assurance, according to UK-based Organisation for the Review of Care and Health Apps (ORCHA).  Social prescribing may support engagement with digital mental health services. |
| Farre et al., 2023 | Use of digital technologies to deliver health services in the UK, focussing on health inequalities. | Evidence for health outcomes for most at-risk groups of health inequalities was lacking in UK. | Authors consider that digital health services in the UK do not work for those most at risk of health inequalities, which is cause for concern considering the increase in the uptake of digital technologies by health services following Covid-19. |
| Peek et al., 2023 | Digital health and care following the pandemic. | Reports rapid increase in digital technologies in the NHS following the pandemic, with concerns that this ultimately opened the door to missed diagnoses, strained therapeutic relationships, and exacerbation of health inequalities. | Greater health inequalities implies greater need for transformation to ensure proper service function in the future.  Suggests that evidence regarding patient-initiated follow up pathways, instead of the traditional physician-initiated model, may result in fewer outpatient appointments but maintain equivalent or better patient satisfaction, quality of life, and clinical outcomes. |

NICE, National Institute for Health and Care Excellence; ORCHA, Organisation for the Review of Care and Health Apps.
